# At-home limbic-targeted transcranial electrical stimulation during sleep: a feasibility study in healthy adults

**DOI:** 10.1098/rstb.2024.0466

**Published:** 2026-09-17

**Authors:** Kyle Morgan, Phan Luu, Roma Shusterman, Damian Persico, Alexei Doncov, Shijing Zhou, Steven Shofner, Don Tucker

**Affiliations:** ^1^ Brain Electrophysiology Laboratory Company, Eugene, OR, USA; ^2^ Department of Psychology, University of Oregon, Eugene, OR, USA

**Keywords:** slow-wave sleep, REM sleep, EEG, TES, limbic cortex, sleep therapy

## Abstract

Deep sleep (N3) and rapid eye movement (REM) sleep are critical for memory and emotional regulation and are often disrupted in neurological and psychiatric disorders. Recent work suggests that focal limbic slow oscillations may provide a target for transcranial electrical stimulation (TES). This study evaluated the feasibility of low-intensity, limbic-targeted TES during sleep and its effects on sleep architecture in healthy adults. A total of 17 healthy participants completed a sham-controlled intervention using an at-home TES device. Stimulation was synchronized with N2 onset, and an overnight electroencephalogram (EEG) was recorded. Sleep stage durations and proportions were compared between TES and off conditions. Limbic-targeted TES was associated with longer total sleep time than the off condition. A repeated-measures ANOVA demonstrated a significant main effect of condition without a significant condition × sleep-stage interaction. Planned pairwise comparisons showed longer absolute N3 and REM sleep durations during TES, whereas only REM increased as a proportion of total sleep time. Participants tolerated the device well, and blinding was maintained. Limbic-targeted TES was feasible to administer during sleep in a natural home environment and was associated with increased total sleep time. These findings provide preliminary evidence of sleep-architecture modulation and support further investigation in larger studies and clinical populations.

This article is part of the discussion meeting issue ‘Digital healthcare for the management of functional neurological disorders’.

## 1. Introduction

Sleep occurs in stages, where each stage plays a role in memory consolidation with varying degrees of importance. Non-rapid eye movement (NREM) stage 3 (deep sleep, or N3) and REM sleep are widely accepted as the two stages that are the most essential for memory consolidation and bodily recovery. N3 sleep is classically identified by large-amplitude slow oscillations (SOs; approx. 0.5–1 Hz) in the human electroencephalography (EEG) record. These synchronized brainwaves facilitate hippocampal–cortical communication and the replay of memory traces that consolidate new experiences into long-term memory [1–3]. REM sleep, although drastically different in the EEG record from N3—characterized by low-amplitude, mixed-frequency EEG that often resembles an awakened state—is functionally complementary to N3 sleep. It facilitates memory processing and the reorganization of large-scale brain networks [4,5]. Recent theoretical work reframes N3 and REM as supporting both the consolidation of unexpected information (N3) and the reinforcement of internal cognitive schemas and expectations (REM, [6]).

The importance of sleep to memory processes is now well demonstrated. It is also clear that sleep disturbances are observed in a wide range of neurological and psychiatric conditions. For example, reduced N3 sleep is associated with impaired glymphatic clearance and an elevated risk for Alzheimer's disease (AD) [7,8], whereas REM sleep fragmentation is commonly found in mood and trauma-related disorders [9,10]. In functional neurological disorder (FND)—a condition characterized by disrupted brain network function without overt structural pathology—disturbances in both N3 and REM sleep are present but understudied. Approximately 58% of FND patients report sleep disruption [11], and more evidence points to altered limbic–cortical connectivity as a key feature [12,13]. Given the overlapping limbic circuitry involved in N3 and REM sleep architecture with FND pathophysiology, improving sleep may present a promising aid in a broader treatment programme. Non-invasive sleep therapy is a current area of active research, with promising approaches using auditory or electrical stimulation to enhance slow-wave sleep [14,15]. Ideally, these interventions should target the underlying neural mechanisms of sleep.

Traditionally, SOs during N3 were understood to reflect travelling waves originating in the frontal neocortex [16,17]. However, there is a competing body of evidence derived from intracranial recordings that suggests a more limbic origin [18,19]. Our previous work using high-density EEG [20] demonstrated that SOs originate from focal discharges in the ventral limbic cortex, specifically the medial temporal lobe and caudal orbitofrontal regions. Using individualized head conductivity models, we identified deep, posterior dipolar inversions that accompany surface-negative frontal potentials—consistent with a limbic origin and more aligned with intracranial recordings [18,19]. These limbic areas are also engaged during REM sleep [21,22], implying that there may be a shared anatomical underpinning between N3 and REM.

Previous studies using transcranial electrical stimulation (TES) applied over the frontal cortex during N3 sleep were able to increase SO power and sleep duration [23,24]. Our lab has extended this approach by applying low-intensity TES (0.5 mA) optimized for targeting the identified anterior *limbic* regions that generate SOs during N3. Using the same electrical head models that allowed identification of the SO sources, we were able to model a set of scalp electrodes (i.e. positions) that represents the optimal current injection pattern for stimulating SO sources [20,25]. This targeted approach allowed us to employ lower TES current levels than prior approaches while still producing significant gains in N3 sleep duration [25]. In this study, we also observed a marginally significant gain in REM sleep duration in response to TES that targets SOs. The gains in N3 and REM sleep are consistent with the importance of the reciprocal regulation of these two sleep stages, with N3 particularly sensitive to unexpected new information and REM important to integrating this new information with the organism's predictive model of the environment [6]. By targeting both phases—either sequentially or adaptively—it may be possible to support more comprehensive consolidation of affective and cognitive processes, especially in disorders involving limbic dysregulation such as FND.

To begin the process of translating this work into a clinical intervention, we developed an at-home TES device capable of delivering low-intensity, limbic-targeted stimulation during sleep. The development of an at-home system would be an important advancement in accessibility and translational potential, allowing individuals to receive neuromodulatory interventions in a natural sleep environment without the need for specialized facilities and continuous supervision. The increased autonomy would also expand the feasibility of conducting large-scale and longitudinal sleep studies.

In this pilot study, healthy adults participated in an at-home, sham-controlled experiment whereby limbic-targeted TES (or sham; referred to hereafter as the ‘Off’ condition) was delivered while they approached deep sleep. We evaluated the feasibility of delivering limbic-targeted TES in an at-home environment using an automated sleep staging and stimulation device and whether TES would increase N3 and REM sleep relative to no stimulation, as has been shown in prior studies.

## 2. Methods

### (a) Participants

Seventeen healthy adult participants (mean age = 27.7, s.d. = 8.8 with a range of 21–53 years old) were recruited for this home-based study based on an *a priori* power analysis (one-tailed repeated-measures design, power 0.8, *d*_z_ = 0.6, alpha 0.05). All participants provided written informed consent prior to participation, and the research protocol was approved by the Institutional Review Board (IRB) at the Oregon Research Institute (Eugene, OR, USA; IRB #00000278, FWA #00005934). Exclusionary criteria consisted of the presence of neurological or psychiatric disorders, sleep apnoea, head injuries, allergies to lidocaine or silver, and self-reported sleep difficulties. Participants were also excluded if they were currently taking medication that would alter their sleep EEG, sleep schedule/habits or reduce their threshold for seizures.

### (b) Experimental protocol

EEG was recorded, and TES (alternating current) was delivered using the wireless interface sensor pod (sleep WISP; Brain Electrophysiology Laboratory, Co., Eugene, OR, USA), a device designed for at-home EEG/TES without the need of a sleep technician. The study consisted of three study sessions, each occurring in the homes of the participants. Before the first night, participants were trained in device setup and application during an orientation session and were supported remotely to ensure proper use at home throughout the protocol. A one-night adaptation session then ensued, consisting of all-night EEG recording (no TES). The night following the acclimation session, participants underwent two nonconsecutive study nights: one with active stimulation and one without (no current delivered, following Marshall *et al*. and Hathaway *et al.* [24,25]), in a counterbalanced order based on subject ID. A one-week washout period was included to minimize potential carryover effects of stimulation between the TES night and Off night.

On each study night, participants applied the WISP headband before bedtime and initiated the recording via a tablet. TES was delivered based on automated, machine-learning classification of sleep stages. Participants and experimenters providing instructions were blinded to condition through the use of nonsensical tablet-entered codes that determined the nightly condition assignment. EEG was recorded throughout the night. Although stimulation intensity was low (0.5 mA) for active nights (TES on) and participants did not wake owing to experienced sensations, there are rare instances (from our prior experience) that some participants can wake owing to felt sensations. To further minimize this possibility and ensure participants could not distinguish between stimulation and the Off condition, use of lidocaine, applied under the stimulation electrodes, occurred on both nights (stim and Off stim). Sixteen out of 17 participants slept through the night as a result (one participant did not utilize lidocaine).

Participants completed standardized sleep and mood questionnaires (e.g. PSQI, ESS, STAI) each morning. Device usage and sleep data were reviewed by research staff for quality control and protocol compliance. All participants successfully completed the protocol and demonstrated proficiency in using the system.

### (c) Wireless interface sensor pod

**Figure 1.**
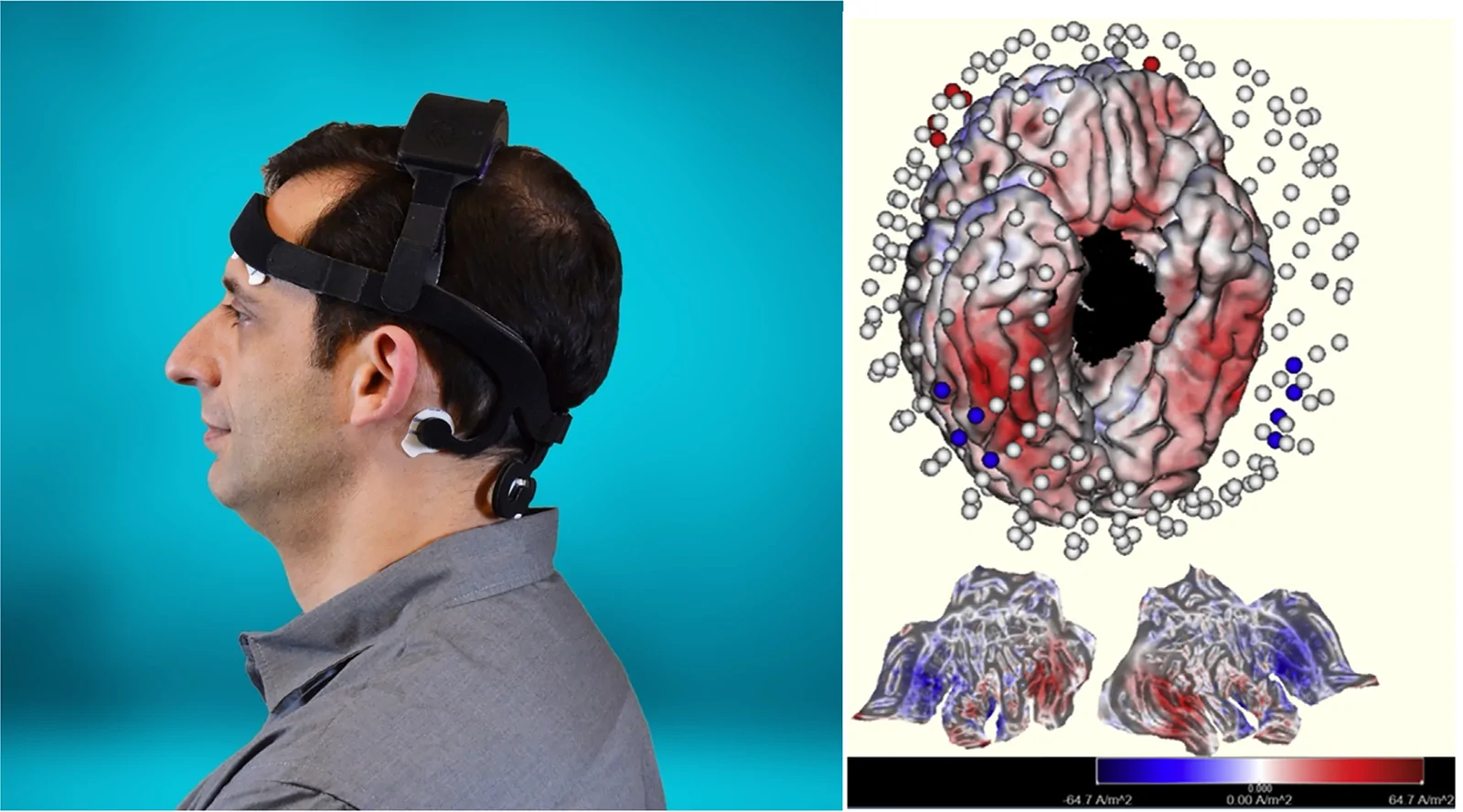
(Left) The sleep WISP headband utilizes electrodes on the forehead, mastoid and back of the neck for EEG recording and TES delivery. An enclosure containing the system's electronics sits on top of the head. The WISP interfaces with a bedside computer (not shown). (Right) A simulation of current flow from the WISP electrode configuration. Current density is highest over ventral limbic cortex (see [25]).

Based on findings that SO sources are localized to medial orbitofrontal and anterior medial temporal regions (see [20]) and TES modelling (using individualized finite difference head models) identified optimal electrode configurations to target these same areas ([25]; figure 1, right), stimulation electrodes of the sleep WISP (Brain Electrophysiology Laboratory, Co.) are at F9 and F10 positions on the forehead and at left and right positions below the hairline at the back of the head. EEG sensors are at frontopolar (Fp1, Fp2) and left and right mastoid positions (see figure 1). All electrodes are 30 × 24 mm in size. TES through the WISP was delivered as a 0.5 Hz sinusoidal (AC) waveform at a maximum amplitude of 0.5 mA (0.25 mA per pair, no ramp-up). Sleep WISP regulates stimulation output to maintain the specified current amplitude despite changes in electrode-to-skin impedance throughout the night. Stimulation was provided in 5-minute blocks separated by 1-minute rest intervals, resulting in 25 total minutes of stimulation on the stimulation night.

The stimulation protocol was applied only once during the stimulation night, and a manual examination of the sleep stages of each participant following the stimulation protocol confirmed that they remained asleep during the protocol. Following the protocol of prior studies that have shown TES enhancement of N3 sleep [24,25], stimulation was initiated approximately 4 minutes after the onset of stage N2 sleep based on real-time sleep staging. No stimulation was delivered on the Off night. EEG through the WISP was recorded at 250 samples per second, and a 0.5–16 Hz bandpass filter was applied prior to sleep scoring.

### (d) Sleep stage classification (NEAT algorithm)

**Figure 2.**
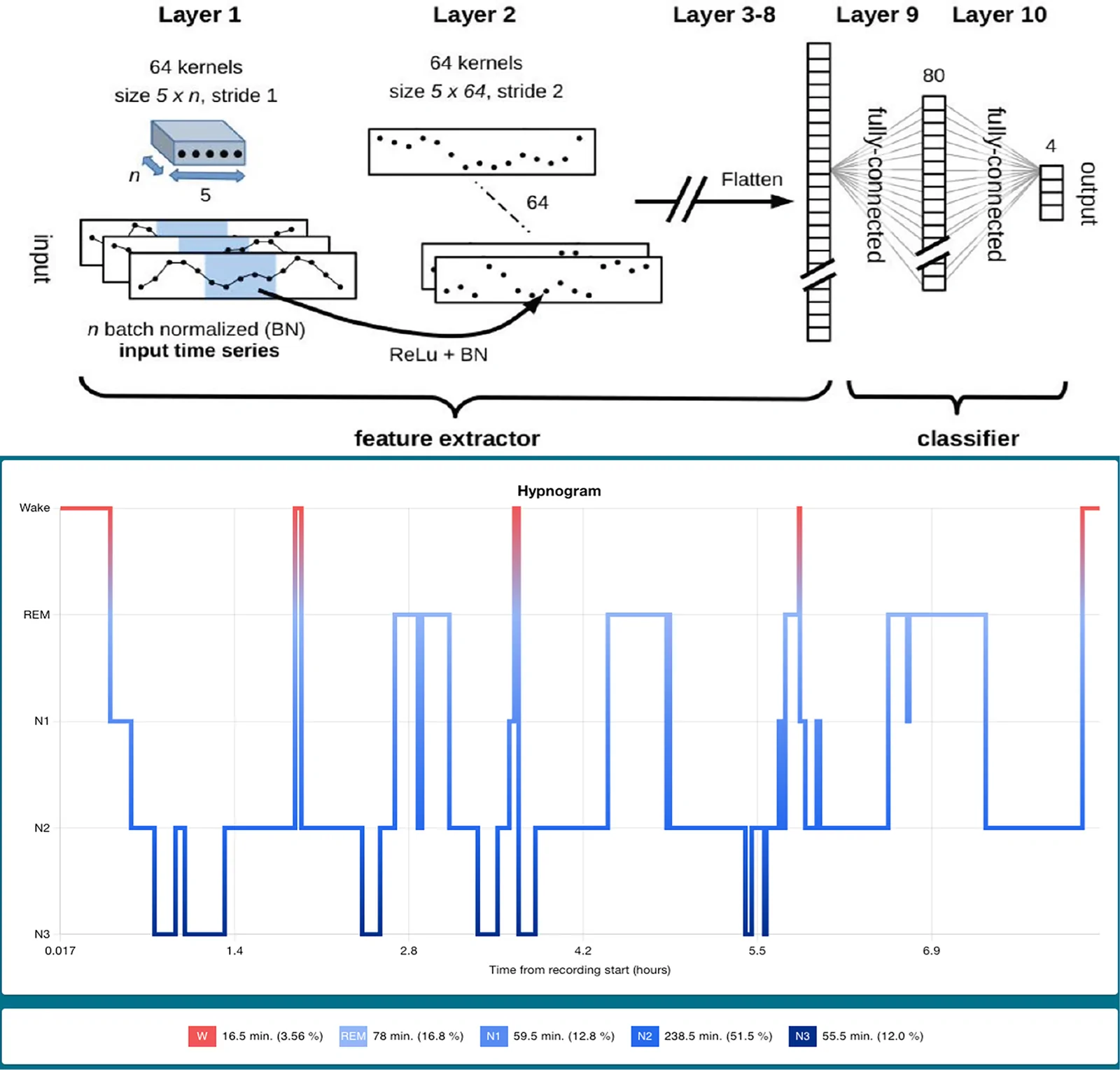
(Top left) The feature extractor within NEAT consists of a hierarchy of eight convolutional layers and produces a feature vector. (Top right) The classifier is composed of two fully connected layers and transforms the feature vector to four class probabilities. (Bottom) An example hypnogram output from NEAT showing the sleep architecture of a single subject from a night without TES.

Sleep staging was performed automatically using a bedside computer running the Neurosom EEG Analysis Technology (NEAT) algorithm, a convolutional neural network (figure 2). NEAT (US FDA cleared as a software medical device (FDA 510(k) K250058)) has been previously validated (for medical device clearance) on 123 overnight polysomnography records (approx. 136 000 thirty-second epochs) spanning participants aged 22–82 years and multiple EEG systems to ensure generalizability across age groups and hardware platforms (see https://www.accessdata.fda.gov/cdrh_docs/pdf25/K250058.pdf). Briefly, the validation was performed as follows: each record was independently scored by three registered polysomnographic technologists, with a two-third majority vote defining the gold-standard label (fewer than 1% of epochs without consensus were excluded); NEAT's performance was then compared to this gold standard using bootstrap statistics (2000 resamples) to estimate sensitivity, specificity and overall agreement with 95% confidence intervals (CIs). Across all epochs, agreement reached 90.3% (95% CI: 90.1–90.6).

### (e) Statistical analysis

All analyses and data processing were conducted using Python version 3.10.11. EEG sleep classification was performed using the NEAT algorithm, and total time spent in each sleep stage—N1, N2, N3 and REM—was calculated for both TES and Off conditions (expressed in minutes). Wake after sleep onset (WASO) was derived by summing all wake events between the first and last three consecutive sleep 30-second events (sleep onset/offset). The total of all sleep events between sleep onset/offset was used to calculate total sleep time (TST) for each session. Sleep stage durations were also expressed as a percentage of TST.

Because of stimulation-induced artefact in the TES night EEG data, all stimulation blocks and the rest periods between blocks were excluded from analysis (29 minutes total). To maintain comparable evaluation of sleep intervals between TES and Off night data (which does not have stimulation artefacts), equivalent-duration intervals were excluded in the Off condition. This was accomplished by excluding data segments at equivalent timepoints (i.e. after the first sign of stable N2 sleep, where TES would have been applied, for 29 minutes).

To evaluate whether the effect of stimulation differed across sleep stages, a two-way repeated-measures ANOVA was conducted using condition (stim, Off) and sleep stage (N1–N3 and REM) as within-subjects factors. Statistical significance threshold was set at *α* = 0.05. Furthermore, based on *a priori* hypotheses derived from prior findings [24,25], that TES produces longer N3 and REM sleep durations but will not affect other sleep stages, planned paired-samples *t*-tests were used to compare the TES and Off conditions for each sleep stage for both raw time (minutes) and a percentage of TST. All tests for REM and N3 were one-tailed based on *a priori* hypotheses predicting increased duration with stimulation. Statistical significance was determined at *α* = 0.05.

## 3. Results

To assess potential order effects, we compared within-subject treatment difference scores (stim minus Off) between participants who received stimulation in the first session versus those who received stimulation in the second session. No significant sequence effects were observed for any sleep stage (Welch *t*-tests all *p* > 0.40). Therefore, we analysed the data according to our *a priori* statistical plan. A series of paired-samples *t*-tests (uncorrected for multiple comparisons because of *a priori* hypothesis prediction) were run to compare changes in sleep architecture between TES and Off conditions. One outlier subject was rejected for analysis based on the mean absolute deviation (MAD) method, which controls for type-I errors [26], leaving 16 subjects for the main analyses.

### (a) Effect of TES on sleep stage durations

**Figure 3.**
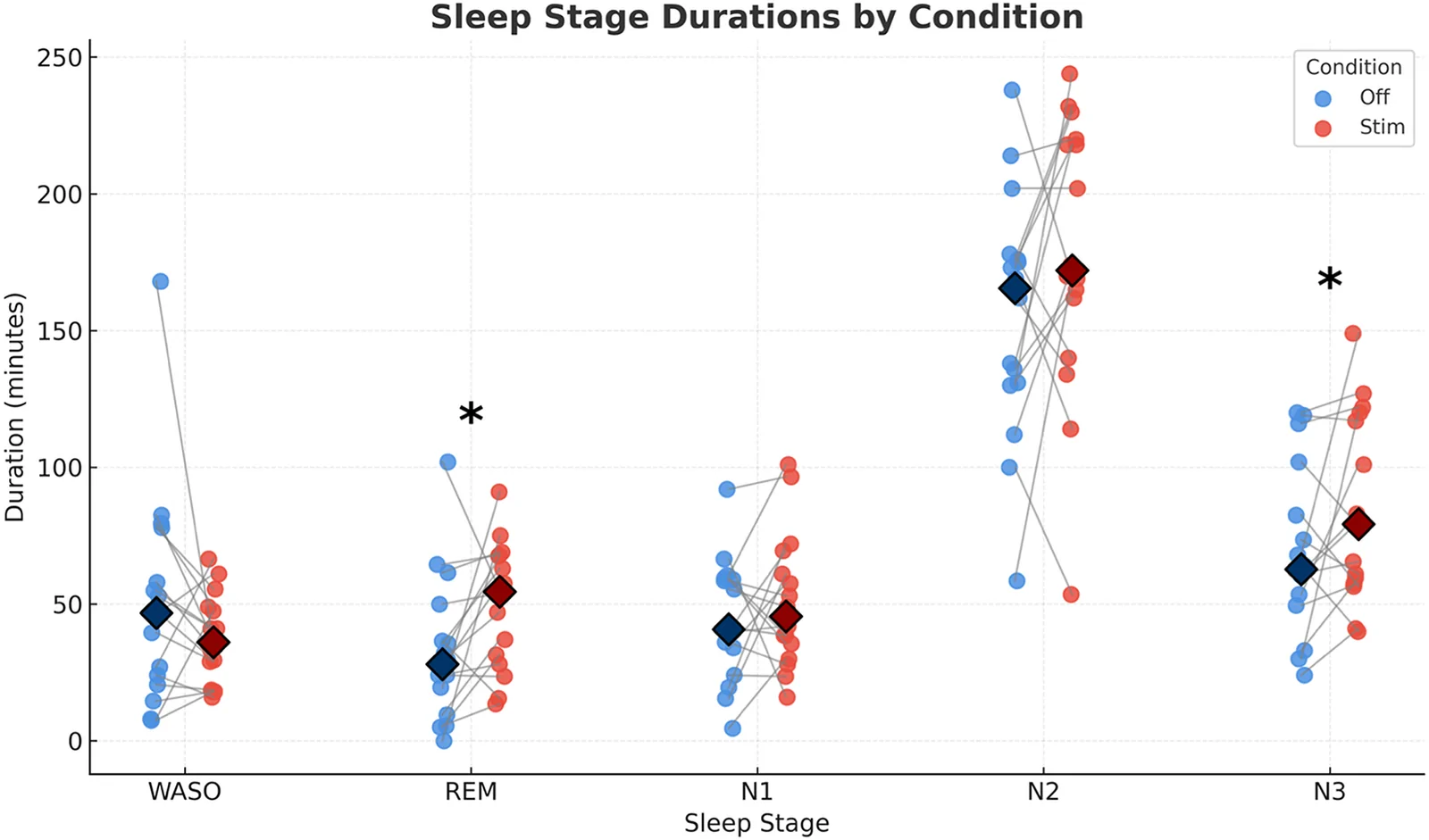
Individual data points are connected with lines to show directionality. A diamond shows the median value for each sleep stage/condition. Subjects spent more time in every sleep stage during the stim night compared to the Off night. An asterisk denotes a statistically significant difference, which was only found for N3 and REM sleep stages.

To evaluate whether TES exerted differential effects across sleep stages, a two-way repeated-measures ANOVA was performed with condition (stim, Off) and sleep stage (N1, N2, N3 and REM) as within-subject factors. A significant main effect of condition was found, *F*(1,15) = 5.91, *p* = 0.028, suggesting participants spent more time asleep overall during the stimulation nights. This was confirmed by pairwise comparison showing that subjects spent significantly more time sleeping overall (TST) during the stimulation night (*M* = 363.1, s.d. = 67.5) than the Off night (*M* = 302.6, s.d. = 81.8), *t*(15) = 2.43, *p* = 0.028 (table 1). There was also a significant main effect of sleep stage, *F*(3,45) = 73.51, *p* < 0.001. However, the condition × sleep stage interaction was not significant, *F*(3,45) = 0.54, *p* = 0.659, indicating that the magnitude of the stimulation effect did not significantly differ across sleep stages. Mean durations (in minutes) for each sleep stage are presented in table 1, and individual data are plotted in figure 3.

**Table 1.** Paired samples *t*-test comparing sleep stage durations, TST and WASO between stim and Off conditions. (One-tailed tests were used for REM and N3 based on directional hypotheses predicting an increase in the stim condition.)

variable	*t*(15)	*p*	test type	mean stim (s.d.)	mean Off (s.d.)
REM	2.25	0.020^*^	one-tailed	49.75 (22.8)	32.81 (26.4)
N1	0.95	0.357	two-tailed	50.47 (24.6)	44.1 (22.3)
N2	1.51	0.151	two-tailed	177.8 (51)	155.8 (45.1)
N3	1.78	0.048^*^	one-tailed	84.94 (33.6)	69.84 (30.8)
TST	2.43	0.028^*^	two-tailed	363.1 (67.5)	302.6 (81.8)
WASO	−1.35	0.196	two-tailed	37.2 (15.7)	50.5 (39.8)

^*^ Significant below 0.05.

To examine TES enhancement of N3 sleep that has been shown in prior research (e.g. [23–25]), planned pairwise comparisons showed that only absolute N3 sleep duration was significantly greater on TES nights (*M* = 84.94, s.d. = 33.6) compared to Off nights (*M* = 69.84, s.d. = 30.8), *t*(15) = 1.78, *p* = 0.048. Although only trending enhancement of TES on REM sleep was observed previously [25], in the present study, participants experienced significantly longer periods of REM sleep (absolute and percentage of TST) on the stimulation night (*M* = 49.75, s.d. = 22.8) compared to the Off night (*M* = 32.81, s.d. = 26.4), *t*(15) = 2.25, *p* = 0.020. No significant differences were found in N1, N2 or WASO durations between the two conditions.

### (b) Sleep stage proportions

**Figure 4.**
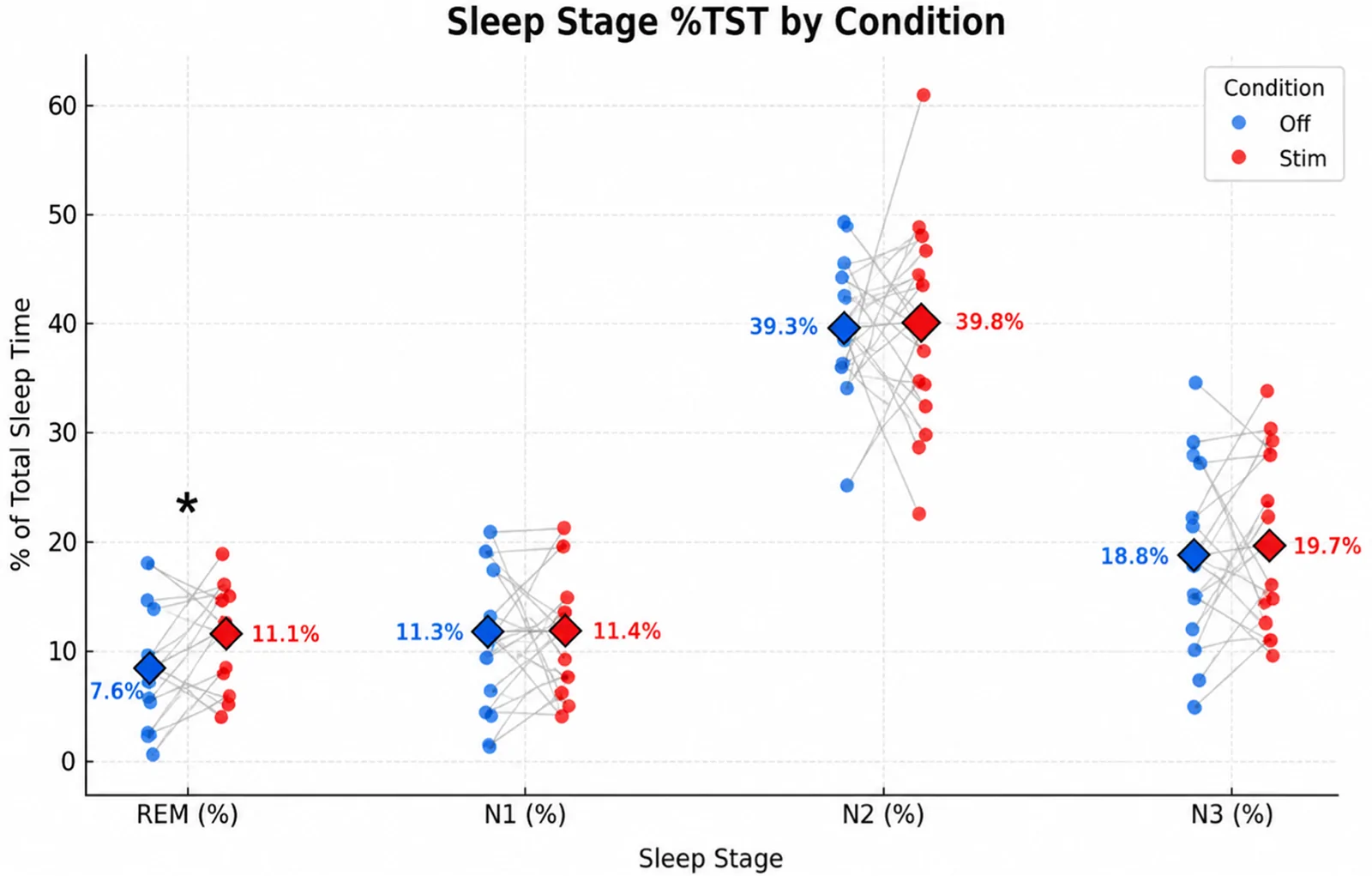
Individual datapoints showing the percentage of total time spent in each stage. Lines show individual trends while a diamond shows the group median. Only the difference in REM reached statistical significance (denoted by an asterisk) where participants spent (proportionally) more time in REM during the stim night compared to the Off night.

Owing to the significant increase in TST during the stimulation night, we compared proportional changes for each sleep stage (figure 4 and table 2). A significant increase was observed only for the proportion of REM in the stim condition (*M* = 11.1%, s.d. = 4.6%) compared to Off (*M* = 7.6%, s.d. = 4.9%), *t*(15) = 2.35, *p* = 0.017, one-tailed. No significant differences were found for N3, N2 or N1 proportions.

**Table 2.** Paired samples *t*-test comparing percentage of sleep stages relative to TST between stim and Off conditions. (One-tailed tests were used for REM and N3 based on directional hypotheses.)

sleep stage	*t*(15)	*p*	test type	mean stim (%) (s.d.)	mean Off (%) (s.d.)
N1% TST	−0.50	0.628	two-tailed	11.4 (4.7)	11.3 (5.2)
N2% TST	−1.11	0.286	two-tailed	39.8 (9.5)	39.3 (9.5)
N3% TST	−0.03	0.513	one-tailed	19.7 (8.3)	18.8 (8.8)
REM % TST	2.35	0.017^*^	one-tailed	11.1 (4.6)	7.6 (4.9)

^*^ Significant below 0.05.

### (c) Acute TES effects

**Figure 5.**
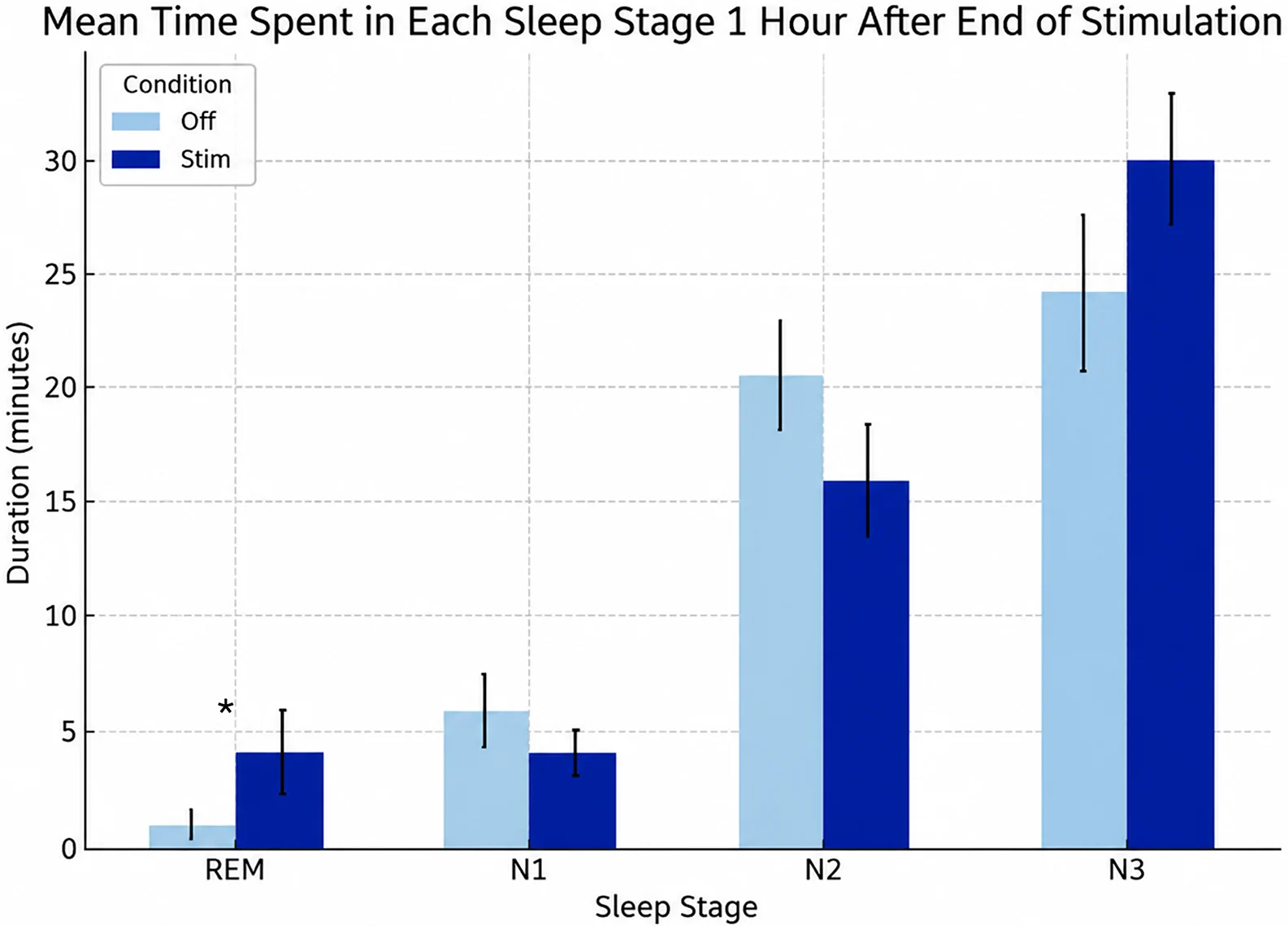
Within one hour of stimulation (or an equivalent window in the Off condition), participants spent longer time in REM and N3 sleep (see text for details). On the stimulation night, N1 and N2 were reduced during this window relative to a comparable window in the Off night.

Based on the observation that TES was associated with longer absolute REM and N3 durations, but increased only REM as a proportion of TST, an analysis was performed to evaluate acute post-stimulation effects. To accomplish this, a one-hour segment of data immediately following the conclusion of TES in the stimulation night and an equivalent period in the Off night was extracted for each subject (figure 5). A paired-samples *t*-test comparing the amount of time spent in each sleep stage during the one-hour post-stimulation (or equivalent in the Off condition) window was performed. As shown in table 3, REM sleep was significantly elevated in the stim condition (*M* = 4.25 minutes, s.d. = 6.1) compared to Off (*M* = 1.00 minutes, s.d. = 2.1), *t*(15) = 1.99, *p* = 0.033, one-tailed. There was also a trend towards increased N3 sleep in the stimulation condition (*M* = 30.28, s.d. = 11.3) relative to Off (*M* = 24.25, s.d. = 13.6), *t*(15) = 1.59, *p* = 0.067, but the effect was not as robust as REM. Additionally, N2 sleep showed a trend towards a reduction in the stim condition (*M* = 15.78, s.d. = 9.2) compared to Off (*M* = 20.63, s.d. = 9.0), *t*(15) = −1.93, *p* = 0.073, two-tailed—suggesting that TES may influence sleep-stage organization during the period immediately following stimulation.

**Table 3.** Paired samples *t*-test comparing sleep stage durations starting one hour after stim/Off. (One-tailed tests were used for REM and N3 based on directional hypotheses predicting increases in deep/restorative sleep following stimulation.)

sleep stage	*t*(15)	*p*	test type	mean stim (min) (s.d.)	mean Off (min) (s.d.)
REM	1.99	0.033^*^	one-tailed	4.25 (6.1)	1.00 (2.1)
N1	−1.35	0.198	two-tailed	3.81 (2.9)	5.72 (5.0)
N2	−1.93	0.073	two-tailed	15.78 (9.2)	20.63 (9.0)
N3	1.59	0.067	one-tailed	30.28 (11.3)	24.25 (13.6)

^*^ Significant below 0.05.

## 4. Discussion

The present pilot study demonstrates the feasibility of delivering limbic-targeted TES during sleep in an at-home setting. Results from the present study show that TES, compared to no stimulation (Off night), produced changes in total time (TST) spent sleeping. Because the order of TES and Off nights was counterbalanced and analysis did not reveal an order effect, the most straightforward explanation for the difference in TST is that longer sleep time as a function of TES reflects sleep-enhancing effects of stimulation across all sleep stages (table 1). Consistent with the overall sleep-facilitating effect of TES is that participants spent less time awake after falling asleep (i.e. WASO was reduced, although not statistically significant) for TES nights.

The repeated-measures ANOVA condition × sleep-stage interaction was not significant. This suggests that TES did not have sleep-stage-specific effects, even though planned *a priori* comparisons revealed longer absolute durations of REM and N3 sleep after TES and only for REM relative to TST. Given the inconsistency of the present result and prior findings of N3 sleep enhancement (e.g. [23–25]), the current data should be interpreted as preliminary evidence that an in-home technology that automatically applies TES during sleep may enhance overall sleep (TST, strongest evidence) and may influence absolute N3 and REM sleep times or only REM sleep time as a percentage of TST.

The overall TST effect observed here, particularly the large difference (approx. 60 minutes), is somewhat surprising. Previous studies with healthy adult populations using stimulation montages with alternating currents similar to Marshall *et al.* [24] show that the effects of TES on sleep enhancement (compared to sham) are highly variable (see [14] for review). There are at least two possible explanations for the large and significant difference we observed in the present study. First, unlike prior studies, the stimulation electrode montage of the WISP was optimized to target sources of N3 SOs [25]. When compared to the montage used by Marshall *et al.*, Hathaway *et al.* showed that the WISP montage impressed higher current density to cortical generators of SOs. This implies that the WISP montage is more effective at stimulating N3 sleep-relevant sources and that this enhancement interacts with REM sleep (see below) to affect TST. Second, the present study, unlike previous studies, was conducted in participants’ homes, where they slept in a familiar environment and also according to their natural bedtimes. Together, these two substantial differences may account for the observed TST, N3 and REM duration enhancements with TES.

Even as the results from these comparisons remain preliminary and need replication in future studies, an important question is why extending N3 sleep results in changes to REM sleep, even though no stimulation was applied during REM, and NREM (including N3) and REM sleep alternate in a way that suggests they are not compatible and may be reciprocally antagonistic. This consideration may also be important to understanding the significant effect of TES on TST, as stimulation during N2–N3 transition appears to affect all sleep stages and particularly so for REM. The answer may reflect the separate forms of homeostatic regulation of NREM and REM sleep, and the intrinsic suppression of REM by the early NREM periods of the night.

The classic two-process model of sleep regulation includes a process S (sleep), which increases sleep pressure with time awake, and a process C (circadian), which varies sleep pressure on a diurnal cycle [27,28]. Process S and NREM sleep pressure are associated with the gradual accumulation of adenosine during the day, causing NREM sleep to be the first sleep stage after sleep onset in normal sleep. REM sleep is regulated by process C (sometimes called process R) in a diurnal fashion, including both brainstem and hypothalamic mechanisms [29]. Furthermore, NREM sleep regulation by the ventrolateral preoptic (VLPO) area of the hypothalamus includes a suppression of REM sleep and other arousal controls while NREM sleep regulation (pressure) is high. The net result is that the progressive transition of normal sleep architecture is characterized by strong and long NREM episodes early in the night, which progressively decrease in SO amplitude and duration over the night, alternating with short REM episodes early in the night (as NREM sleep pressure gradually declines) and increasing REM strength and duration as NREM sleep declines and REM pressure continues to be manifested [3].

As a result of these dynamics, it appears that the present technique of applying TES after the first stable N2 episode not only changes the duration of the following N3 episode, but more rapidly depletes the NREM pressure, decreasing the VLPO suppression of multiple arousal controls, and thereby releasing REM from inhibition. Although further research is required to understand the mental health implications of this specific intervention, there appear to be therapeutic benefits not only in influencing NREM consolidation but also in modulating REM sleep, with potential benefits for emotional integration [5,30].

As noted, an important difference of the present study compared to prior studies is the setting in which the study was conducted. Sleep studies are inherently time-consuming, requiring lab personnel, time commitment from participants to go to the lab etc., and they also present a foreign sleep environment for participants. These limitations have implications not only for studies (e.g. limited number of participants owing to cost and inconvenience) but also for the impact of any future clinical application.

The significance of these findings for FND suggests the feasibility and possibility of significant sleep improvement with a simple, at-home, neuromodulation device. Little is known about sleep disorders in FND. The most comprehensive review of FND and sleep disturbances is the recent structured literature review following the PRISMA guidelines performed by Kannan *et al.* [11]. In this review, it was found that some form of sleep dysfunction was reported in 58% of patients with FND. How such sleep deficits may contribute to FND, and the underlying symptoms, are not well understood at this time. Although our results are not immediately relevant to understanding the underlying mechanisms of FND and sleep, they do suggest that a simple and powerful artificial synchronization of the SOs of N3 impacts both the N3 stage and the subsequent durations of REM sleep. This in turn may be relevant to understanding FND as well as potential therapy when it becomes clear how FND and sleep may be mechanistically related.

## Data Availability

Reviewers may access the data for this article through Zenodo [31].

## Theme

One contribution of 12 to a discussion meeting issue ‘Digital healthcare for the management of functional neurological disorders’.
